# Cancer Risk Communication in Mainstream and Ethnic Newspapers

**Published:** 2008-12-15

**Authors:** Jo Ellen Stryker, Jessica Fishman, Karen M. Emmons, Kasisomayajula Viswanath

**Affiliations:** Behavioral Sciences and Health Education, Rollins School of Public Health, Emory University; University of Pennsylvania, Philadelphia, Pennsylvania; Dana-Farber Cancer Institute and Harvard School of Public Health, Boston, Massachusetts; Dana-Farber Cancer Institute and Harvard School of Public Health, Boston, Massachusetts

## Abstract

**Introduction:**

We wanted to understand how cancer risks are communicated in mainstream and ethnic newspapers, to determine whether the 2 kinds of newspapers differ and to examine features of news stories and sources that might predict optimal risk communication.

**Methods:**

Optimal risk communication was defined as presenting the combination of absolute risk, relative risk, and prevention response efficacy information. We collected data by conducting a content analysis of cancer news coverage from 2003 (5,327 stories in major newspapers, 565 stories in ethnic newspapers). Comparisons of mainstream and ethnic newspapers were conducted by using cross-tabulations and Pearson χ^2^ tests for significance. Logistic regression equations were computed to calculate odds ratios and 95% confidence intervals for optimal risk communication.

**Results:**

In both kinds of newspapers, cancer risks were rarely communicated numerically. When numeric presentations of cancer risks were used, only 26.2% of mainstream and 29.5% of ethnic newspaper stories provided estimates of both absolute and relative risk. For both kinds of papers, only 19% of news stories presented risk communication optimally. Cancer risks were more likely to be communicated optimally if they focused on prostate cancer, were reports of new research, or discussed medical or demographic risks.

**Conclusion:**

Research is needed to understand how these nonnumeric and decontextualized presentations of risk might contribute to inaccurate risk perceptions among news consumers.

## Introduction

The news media play a role in educating the public about cancer risks (1-3). In turn, cancer risk perceptions influence cancer-related health behaviors, according to established behavioral theories, such as the Health Belief Model (4), the Extended Parallel Process Model (5), and the Precaution Adoption Model (6). These models state that behavior change is likely when a person believes that a given behavior poses nontrivial risks and when that person has self-efficacy. In addition to these conceptual frameworks, many empiric studies (7,8) have suggested that holding beliefs that a given behavior poses nontrivial risks is a necessary (though not necessarily sufficient) condition for changing related behaviors (5) and provoking policy change (9).

Mass media communication, as well as personal experience and interpersonal communication, shape our perceptions of risk. Although each source is influential, media exposure is particularly influential under certain conditions (10). For instance, mass media communication can be particularly influential when it is a primary source for health information, when direct experience is limited, or when perceptions of risk are low (11,12).

The amount and type of media attention paid to particular risks influence risk perceptions. The salience of risk perceptions is highly associated with the extent of news coverage of instances of those risks (13,14). Furthermore, media attention to a particular risk may contribute to a social amplification of risk perception (10). Previous studies have also evaluated the quality of risk communication in news media. The news media have played a positive role by educating the public about health risks and influencing behavior change (15,16), but the reporting often includes inadequate or incomplete information about risks (17-19).

The interpretation of risk by physicians, consumers, and third-party payers may be influenced by incomplete presentations of risk (20-23). For instance, reporting relative risk alone (without clearly specifying event rates) leads many to overestimate the magnitude of findings (22). Alternatively, reporting absolute risk alone may lead to an underestimate of risk perceptions, particularly for groups at higher risk. Because the reporting of risk can be misleading, the news media and other health communicators have been urged to provide absolute risk or both relative and absolute risks when quantifying risks (24-26).

Newspapers have been repeatedly cited by survey respondents as a trusted source of health information (1,27,28). Because newspapers reach distinct segments of society (29), whether quality risk communication is given to all groups should be monitored. Therefore, we evaluated cancer risk communication in newspapers targeting mainstream and ethnic audiences (those who share a country of origin, religion, or race). A recent poll showed that 45% of adults from ethnic minority groups surveyed preferred ethnic media to mainstream media, and an additional 35% accessed ethnic media regularly (29). Culturally specific messages may resonate more with certain audiences and ultimately be more persuasive (30,31).

Cancer news coverage might differ between mainstream and ethnic newspapers for several reasons. Mainstream media organizations are generally larger, more likely to report scientific research, and have the resources to support trained medical and science reporters. Smaller newspapers are more likely to report human interest stories and to lack the resources to support medical and science reporting (32,33). Moreover, ethnic media serve a different role than do mainstream media. Ethnic media producers believe their role is to focus on culturally distinct aspects of the news (30,31). Furthermore, given the homogeneity of their audiences, ethnic newspapers may be more likely to discuss health risks that are highly salient to their readers. Alternatively, certain beliefs that are widely held among particular ethnic groups, such as cancer fatalism (34) or the taboo nature of discussing cancer, may influence cancer portrayals in ethnic media.

Cancer news coverage may influence cancer-related behavior. For example, news coverage can produce short-term increases in attention to particular issues, such as an increase in screening rates when a celebrity is diagnosed with cancer (35). Alternatively, it can influence long-term secular trends in behaviors such as smoking (36). However, little research has examined how cancer coverage may affect cognitive and psychosocial factors such as cancer risk knowledge or perceptions (37). Our research attempts to fill that gap by examining how newspapers cover cancer risks.

The purpose of this study was to explore the frequency of articles about different types of cancer risks that are published by mainstream and ethnic newspapers, to assess the extent to which newspapers communicate cancer risks optimally, and to determine whether portrayals differ between the 2 kinds of newspapers. Given that few empiric tests of optimal risk communication (ORC) messages have targeted large audiences, the understanding of ORC strategies is limited. Our definition of ORC is derived from areas that appear to have a consensus: 1) that risk should be placed in context by providing both absolute and relative risk and 2) that discussions of risk may instill perceptions of threat, which could trigger reaction or denial if they are not countered with some discussion of response or prevention efficacy.

We also determined whether key story features were associated with ORC. Stories about particular types of cancer or particular types of cancer risks may be more likely to present cancer risks optimally. Since larger news organizations such as wire services have greater resources and trained health reporters, stories from a wire service may be more likely to use ORC. The same may be true for stories using expert sources. Finally, differences in ORC may be attributable to the basic story formats we identified for cancer news articles. For example, a profile of a person dealing with cancer may be more focused on developing a compelling narrative than on providing ORC. In contrast, a report of new research may be more likely to focus on scientific information and, hence, to communicate risk optimally.

We asked what types of cancer risks are discussed most frequently and whether mainstream and ethnic newspapers differ; how often cancer risks are communicated optimally in mainstream and ethnic newspapers; and whether certain features of the story or source are predictive of ORC in both mainstream and ethnic newspapers.

## Methods

A content analysis was conducted using US mainstream and ethnic papers for 2003. We defined "mainstream" as the 50 highest-circulation newspapers that were accessible through the Lexis-Nexis database (n = 44). We defined "ethnic" as all English-language newspapers in the Ethnic NewsWatch database (n = 283), representing many racial and ethnic groups, including African American, Native American, and Latino.

We used a 2-stage process and a combination of electronic and manual methods to retrieve valid stories. The fundamental criterion for validity was that stories contain a minimal amount of cancer information (approximately 2 sentences or more). The first stage, described in greater detail elsewhere,  entailed developing a search term that filtered out irrelevant stories without removing relevant stories (38). The final search term retrieved stories that mentioned cancer, pseudonyms for cancer (eg, tumor), and specific types of cancer at least twice, while ruling out irrelevant references to cancer such as in the context of horoscopes (ie, cancer as an astrological sign). The search term retrieved 26,784 stories from the mainstream papers and 696 from the ethnic newspapers. Stories from the mainstream newspapers were entered into a database and randomly retrieved for review (n = 9,154), and all ethnic stories underwent review.

In the second stage, 4 coders manually reviewed the stories. Valid stories were coded for a range of variables. Coders received approximately 90 hours of training over 4 months before conducting intercoder reliability tests, and reliability was rechecked every 3 months after coding began. Tests were conducted on samples of approximately 150 stories from mainstream newspapers that appeared in the months adjacent to the study period. The overall content analysis used many measures (39), but this report focuses on the risk communication variables and variables that might be predictive of ORC. All variables included in this report had a mean Krippendorff's α > .60.

The number of variables coded depended on the quantity of cancer information in a story, classified as minimal (approximately 2 sentences), major theme (approximately 3 sentences or more but not the primary focus of the article), or primary focus. The overall sample contained 5,327 mainstream and 565 ethnic newspaper stories that had a minimal amount of cancer information. However, only the stories for which cancer was a major theme were coded for risk communication variables; hence, the sample for analyses presented here is limited to this subset of stories (3,638 mainstream, 380 ethnic).

### Measures

We analyzed 42 measures. A general measure of morbidity risk evaluated whether an article included any discussion of cancer morbidity risk, either implicitly (eg, by discussing smoking in an article without explicitly stating that smoking is a cancer risk factor) or explicitly by discussing the chances of developing cancer. These morbidity stories were then coded for whether they quantified the risk numerically.

We noted the presence or absence of 5 general types of risks: lifestyle, genetic, demographic, medical, and environmental/occupational. Within the general types of risk, 17 specific cancer risks were assessed. For lifestyle risks, we measured alcohol and tobacco use, exercise, diet, sexual practices, sun exposure, and obesity. Demographic risks included race, age, and socioeconomic status (SES). Environmental/occupational risks included air or water pollutants, pesticides/chemicals, and occupational hazards. Medical risks included medications, surgery, and viruses or other infectious agents. Response options for the specific risks included not mentioned, implicit (eg, Sheila, a smoker, died from lung cancer), explicit but nonnumerical (eg, smoking causes lung cancer), or numerical (eg, smokers are 11 times more likely to develop lung cancer than nonsmokers). We did not measure any subset of genetic risk but did specify the format of risk presentation. The implicit and explicit but nonnumeric categories were subsequently collapsed into 1 category representing nonnumeric risk.

The ORC variable comprised 2 general sets of measures: numeric risk and prevention response efficacy information. We coded every story that contained a numeric presentation of morbidity risk for the presence or absence of absolute and relative risk. From these measures, we computed a variable to measure whether both types of risk were presented in the same story. We used 3 measures of response efficacy: 1) information about prevention, 2) information about screening, or 3) mobilizing information (ie, resources provided for readers to obtain additional cancer information). For stories that used numeric presentations of risk and presented either absolute or relative risk, we computed an ORC variable. We classified stories that discussed both absolute and relative risk and provided some type of efficacy information as ORC stories. We classified stories that provided either absolute risk or relative risk but not both, or did not provide efficacy information, as not being ORC stories.

We also measured features of the stories that might be associated with ORC, which fell into 5 categories. First, we examined the type of cancer being discussed, which we limited to the 6 cancers with the highest incidence rates: lung, prostate, colorectal, breast, skin, and female reproductive. Second, we examined the general categories of risk discussed above. Third, given that wire services tend to have specialized health reporters and thus may be more likely to present ORC, we considered whether the story originated from a wire service. Fourth, we looked at the relationship between whether certain sources or types of sources were cited and ORC. In particular, we looked at the 2 most widely known cancer organizations, the National Cancer Institute and American Cancer Society, as well as the general categories of scientific journals, research institutions, and pharmaceutical companies. Other cancer or government organizations were not coded because acceptable measures of reliability could not be achieved. Finally, we considered whether ORC varies based on the format of the story. Format categories included reports of new research, profiles of people dealing with cancer, awareness and education, policy and politics, fundraisers, and other.

### Analysis

Mainstream and ethnic newspapers were compared by using cross-tabulations and Pearson χ^2^ tests for significance. To determine variations in the presentation of ORC, logistic regressions were computed to calculate odds ratios (OR) and 95% confidence intervals (CIs). Initially, bivariate analyses were conducted. Distinct constructs were entered separately and related variables entered jointly. Any variable that was significant at the bivariate level was included in a multivariate model.

## Results

Overall, the ethnic newspapers were substantially more likely than the mainstream newspapers to report on morbidity risks (mainstream, 39.3%; ethnic, 61.6%; χ^2^ = 70.15, *P* < .001).

Each of the general types of risks discussed was significantly different between mainstream and ethnic newspapers  (Table 1). When morbidity risk was discussed, more ethnic than mainstream newspapers discussed demographic risks. Conversely, although medical cancer risk discussions were less common than other risk types, mainstream newspapers discussed these risks more than twice as often as ethnic newspapers. More mainstream newspapers discussed occupational or environmental cancer risks, and more ethnic newspapers presented genetic cancer risks. Lifestyle risks were commonly discussed in both types of newspapers.

Numeric descriptions of specific cancer risks were infrequent (Table 2). Summing numeric descriptions of risk across the specific cancer risks discussed, only 29% of ethnic newspaper and 21% of mainstream newspaper descriptions of specific risks were expressed numerically (χ^2^ = 6.84, *P* < .001). More mainstream newspapers discussed cancer morbidity risks related to medications, air or water pollutants, and occupational hazards. More ethnic newspapers discussed cancer morbidity risks related to genetics, physical activity, diet and nutrition, obesity, and age. The largest difference in risk reporting was race/ethnicity, which was mentioned as a risk factor in 51% of ethnic stories but in only 11% of mainstream stories.

Numeric descriptions of cancer morbidity risks overall were more common than numeric descriptions of specific cancer risks. Among stories discussing cancer morbidity risks (1,431 mainstream, 234 ethnic), approximately 42% of mainstream and 52% of ethnic newspapers provided some numeric presentation of cancer morbidity risks (χ^2^ = 7.71, *P* < .01). Although more ethnic newspapers provided numeric presentations of risk, no significant differences were seen between the 2 news sources in the numeric formats used to describe risk. When numeric presentations of risk were provided (mainstream n = 607, ethnic n = 122), absolute risk was discussed in 71% of mainstream newspapers and 72% of ethnic papers. Relative risk was less commonly discussed, appearing in 40.9% of mainstream and 37.7% of ethnic newspapers. Few stories placed risk in context by presenting both absolute and relative risk in the same story: only 26.2% of mainstream and 29.5% of ethnic stories that used numerical formats to describe cancer morbidity risks provided estimates of both absolute and relative risk.

Among stories discussing cancer morbidity risks, more ethnic newspapers provided response efficacy information. No significant difference was seen between the 2 news sources in the likelihood of providing prevention information, but more ethnic media provided both screening and mobilizing information. However, any differences in the provision of efficacy information in the context of cancer risk may be an artifact. Among all cancer stories, not just those discussing risk, more ethnic newspapers than mainstream newspapers presented all 3 types of efficacy information (Table 3). Thus, the greater tendency to provide efficacy information in cancer risk stories may be a reflection of the greater overall tendency of the ethnic newspapers to provide efficacy information in cancer stories.

Looking only at stories that provided either absolute or relative risk information (566 mainstream, 108 ethnic), few stories provided risk in context as well as efficacy information. Only 19% of stories overall presented ORC. There was no significant difference between mainstream and ethnic newspapers with respect to ORC (Table 4).

The only significant difference in ORC based on story format was that, compared with an "other" category, reports of new research were 2.36 times more likely to provide an ORC. Stories clearly originating from a wire service were 1.61 times more likely to communicate risk optimally compared with local stories or stories of an unspecified origin. Of the 6 most commonly occurring cancers, only stories discussing prostate cancer were more likely to present risk optimally. Across the 3 general and 2 specific sources cited in cancer news stories, stories that cited the American Cancer Society or scientific journals were more likely to present risk optimally, while stories citing the National Cancer Institute, research institutions, or pharmaceutical companies were not predictive of ORC. Of the 5 categories of risk, stories discussing lifestyle, demographic, and medical risks were more likely to present risk optimally than stories not discussing those particular risks. ORC was most likely to be found in stories discussing demographic risks: these stories were 2.55 times more likely to present risk optimally than stories that did not discuss demographic risks.

In a multivariate model, reports of new research, reliance on a wire service, citing the American Cancer Society, and discussing lifestyle risks were no longer significant predictors of ORC. The other significant predictors remained. Stories that discussed prostate cancer, cited a scientific journal, discussed demographic risks or medical risks were more likely to discuss risk optimally, by presenting both absolute and relative risk and some response efficacy information.

## Discussion

Cancer morbidity risks are commonly presented in cancer news stories. In both mainstream and ethnic newspapers, the magnitude of specific risk factors was rarely communicated numerically. When numeric presentations of cancer risks were used, newspaper articles tended to present either absolute or relative risk rather than contextualize the magnitude of risk by presenting both absolute and relative risk in the same article. Thus, few news stories presented an optimal communication of risk by including both absolute and relative risk as well as providing some type of efficacy information. Cancer risks were more likely to be communicated optimally if they focused on prostate cancer, were reports of new research, or discussed medical or demographic risks. The finding that prostate cancer risks were most likely to be communicated optimally requires more investigation. One possible explanation is the level of uncertainty and controversy regarding prostate cancer screening and treatment, which might encourage reporters to try to offer background information. As a result, more risk and response efficacy information appears in these news stories.

Across both news sources, lifestyle and demographic risks were the risk types most often mentioned in cancer news stories. There were striking differences in many of the specific cancer risks discussed. The ethnic newspapers were only slightly more likely to discuss lifestyle risks in general, but they were much more likely to discuss specific lifestyle risks, including risks related to tobacco, physical activity, diet or nutrition, and obesity.

This study has several limitations. First, this analysis is limited to cancer news that appeared in print newspapers and is not meant to be representative of cancer news coverage in other communication channels. Nonetheless, while the specific content of news stories may vary across media formats as a function of time, space, and other institutional normative differences, the topics being covered in newspapers and television are often similar (40).

Differences in format between the ethnic and mainstream newspapers might have affected the results. For example, the mainstream newspapers were all printed daily, but the ethnic sample included newspapers that were printed less often. We have no way of knowing whether the differences in publication frequency could account for the observed differences in the cancer topics covered across 2 newspaper sets. The mainstream and ethnic news samples were not matched based on the city of publication, thus introducing the possibility that regional differences could be responsible for any observed differences between the 2 newspaper types.

The ethnic papers in this study were limited to English-language papers because Ethnic NewsWatch only archives newspapers printed in English or Spanish. Although we cannot be certain the inclusion of ethnic papers printed in native languages would have altered the results, we did compare stories from Latino newspapers printed in English and Spanish and found that the coverage was similar.

Finally, we recognize the limitations inherent in using a generic classification of ethnic newspapers, a necessity based on sample size. Although ethnic newspapers share some features in common, ethnic groups differ with respect to cancer risks. By grouping all ethnic newspapers together, any such differences in newspaper coverage are lost. Further research among distinct ethnic media is needed to understand these differences.

Despite these limitations, this study contributes to our understanding of how cancer risk information is presented to the public by newspapers, which has implications for how the public may learn about cancer risk information. Information about cancer risks, in general, lacked context. Less than half of newspaper stories that discussed cancer risks contained any type of efficacy information, and typically presented risks using nonnumeric formats. This lack of contextual information, which is consistent with other studies that have examined the context of risk information in the mass media (10,41), may explain why news coverage is more likely to activate societal rather than individual risk perceptions. These nonnumeric descriptions, coupled with a general lack of contextual information, may make it difficult for readers to properly evaluate their own cancer risks (10). Readers may understand that something poses a cancer risk, but in the absence of concrete information, choose to believe that the risks are only relevant for other people.

Results from this study, other published data from this content analysis (39), and emerging research (42) suggest that ethnic newspapers may have a stronger commitment to cancer prevention and education than do mainstream newspapers. Although newspaper stories about modifiable cancer risks may not conform to traditional journalistic standards for newsworthiness (32,43,44), ethnic newspapers serve a different role than mainstream media. Given the homogeneity of their audiences, ethnic media can be sensitive to any threats to the ethnic community and try to disseminate any information that can ameliorate that threat (45). This commitment, coupled with the fact that ethnic media can communicate in a culturally and linguistically appropriate manner, suggests the utility of engaging ethnic media in health promotion efforts. One way to increase the likelihood of generating media coverage in ethnic media may be to frame health concerns in the context of risk for the particular ethnic group served by the newspaper. Given the homogeneity of audiences, the potential for creating unintended consequences, such as increased stigma among people who are not a part of the group, is low, yet the potential for generating increased media coverage is great.

## Figures and Tables

**Table 1:** Risk Categories Described in Stories About Cancer Morbidity Risk, by Newspaper Source, 2003

Risk Category	Stories Overall (N = 1,665), %^a^	Stories in Mainstream^b^ Newspapers (n = 1,431), %^a^	Stories in Ethnic^c^ Newspapers (n = 234), %^a^	*P* value^d^
Lifestyle	41.0	40.3	47.9	.03
Genetic	20.0	18.7	28.2	.001
Demographic	35.6	30.9	64.1	<.001
Medical	16.9	18.4	7.3	<.001
Environmental/occupational	19.9	21.2	12.0	<.001

a Columns do not total 100% because some stories mention more than 1 risk category.

b "Mainstream" was defined as the 50 highest-circulating newspapers that were accessible through the Lexis-Nexis database (n = 44).

c "Ethnic" was defined as all English-language newspapers in the Ethnic NewsWatch database (n = 283).

d *P* values were calculated by using Pearson χ^2^ analysis.

**Table 2:** Percentage of Stories About Cancer Morbidity Risk That Mention Specific Cancer Risks and Formats^a^ Used to Describe the Risks, by Newspaper Source, 2003^b^

Cancer Risk Category	Mainstream Newspapers^c^			Ethnic Newspapers^d^			*P* Value^e^
	**Overall^f^**	**Numeric^f^**	**Nonnumeric^f^**	**Overall^f^**	**Numeric^f^**	**Nonnumeric^f^**	
**Lifestyle**							
Alcohol	3.6	0.5	3.0	6.4	0.4	6.0	.09
Tobacco	18.4	1.7	16.6	23.5	4.3	19.2	.02
Exercise	5.4	0.5	4.9	14.5	0	14.5	<.001
Diet/nutrition	14.3	1.5	12.7	31.2	1.3	29.9	<.001
Sexual practices	2.4	0.3	2.2	2.6	0	2.6	.67
Sun exposure	6.6	2.7	4.0	3.0	0.4	2.6	.06
Obesity	5.5	0.7	4.8	11.1	0	11.1	<.001
**Genetic**	18.7	2.0	16.7	28.2	3.0	25.2	.003
**Demographic**							
Race	11.0	3.4	7.6	51.3	17.5	33.8	<.001
Age	22.8	2.4	20.3	29.1	1.3	27.8	.02
SES	4.2	0.3	3.8	3.0	0.4	2.6	.61
Medications	12.4	3.6	8.7	2.6	0	2.6	<.001
Surgery	3.1	0.3	2.8	2.1	0	2.1	.56
Virus/infectious agent	3.8	0.7	3.1	2.1	0	2.1	.32
**Environmental/occupational**							
Air/water pollutants	9.8	0.9	8.9	4.7	0.4	4.3	.04
Pesticides/chemicals	8.7	0.8	7.9	5.6	0	5.6	.16
Occupational hazards	7.3	0.6	6.6	1.3	0	1.3	.002

Abbreviation: SES, socioeconomic status.

a "Numeric" refers to story formats that quantified cancer risk; "nonnumeric," to story formats that did not quantify cancer risk.

b Because of rounding, numeric and nonnumeric percentages may not equal overall percentages.

c "Mainstream" was defined as the 50 highest-circulating newspapers that were accessible through the Lexis-Nexis database (n = 44).

d "Ethnic" was defined as all English-language newspapers in the Ethnic NewsWatch database (n = 283).

e *P* values were calculated by using Pearson χ^2^ analysis.

f Percentage values do not necessarily correspond with Table 1 because some stories contain more than 1 risk category.

**Table 3:** Type of Response Efficacy Information in Stories About Cancer Morbidity by Newspaper Source for Cancer Stories Overall and for Cancer Morbidity Risk Stories Only, 2003

Type of Story/Response Efficacy	No. of Overall Stories (%)	No. of Mainstream^a^ Stories (%)	No. of Ethnic^b^ Stories (%)	***P* Value** c
**Among stories discussing morbidity risk (n = 1,665)**				
Prevention information	48.4	48.1	50.4	.51
Screening information	32.4	29.9	47.4	<.001
Mobilizing information	19.8	18.6	26.9	.003
**Among all stories with a major cancer theme (N = 4,018)**				
Prevention information	20.8	19.6	32.4	<.001
Screening information	20.4	18.8	35.3	<.001
Mobilizing information	17.5	16.8	23.9	<.001

a "Mainstream" was defined as the 50 highest-circulating newspapers that were accessible through the Lexis-Nexis database (n = 44).

b "Ethnic" was defined as all English-language newspapers in the Ethnic NewsWatch database (n = 283).

c *P* values were calculated by using Pearson χ^2^ analysis.

**Table 4:** Predictors of Optimal Risk Communication^a^ in Newspaper Stories About Cancer Morbidity Risk, 2003

**Variable**	No. of Stories (%)	Optimal Risk Communication		

		%	OR (95% CI)	AOR (95% CI)
**News type (reference: mainstream)**				
Ethnic	108 (16.0)	22.2	1.27 (0.77-2.10)	NC
**Story format (reference: other)**				
Report of new research	344 (51.0)	23.3	2.36 (1.13-4.93)	1.09^c^ (0.63-1.90)
Policy/politics	47 (7.0)	17.0	1.60 (0.57-4.47)	NC
Awareness/education	118 (17.5)	17.8	1.68 (0.73-3.90)	NC
Profile	86 (12.8)	11.6	1.02 (0.39-2.67)	NC
**Origin (reference: not wire service)**				
Wire service	134 (19.9)	25.4	1.61 (1.03-2.53)	1.15 (0.68-1.95)
**Cancer type (reference: not cancer type)^b^**				
Lung	124 (19.1)	24.2	1.31 (0.79-2.20)	NC
Breast	288 (44.4)	20.5	1.21 (0.79-2.20)	NC
Colorectal	125 (19.3)	20.8	.92 (0.54-1.58)	NC
Skin	112 (17.3)	25.0	1.54 (0.94-2.53)	NC
Prostate	149 (23.0)	28.2	1.88 (1.21-2.91)	1.90 (1.18-3.05)
Female reproductive	102 (15.7)	19.6	.99 (0.56-1.73)	NC
**Sources cited (reference: source not cited)^b^**				
National Cancer Institute	100 (14.8)	27.0	1.39 (0.83-2.31)	NC
American Cancer Society	186 (27.6)	28.0	2.05 (1.35-3.09)	1.49 (0.95-2.33)
Scientific journals	215 (31.9)	28.4	2.12 (1.39-3.25)	1.78 (1.05-3.03)
Research institutions	345 (51.2)	22.3	1.17 (0.77-1.80)	NC
Pharmaceutical companies	48 (7.1)	22.9	1.12 (0.54-2.32)	NC
**General risk type (reference: not risk type)^b^**				
Lifestyle risk	256 (38.0)	25.4	1.54 (1.02-2.33)	1.54 (1.00-2.35)
Genetic risk	160 (23.7)	26.3	1.12 (0.71-1.78)	
Demographic risk	306 (45.4)	27.8	2.55 (1.66-3.92)	2.06 (1.31-3.23)
Medical risk	143 (21.2)	31.5	2.29 (1.47-3.56)	2.17 (1.34-3.50)
Environmental/occupational risk	99 (14.7)	22.2	1.43 (0.82-2.48)	NC

Abbreviations: OR, odds ratio; CI, confidence interval; AOR, adjusted odds ratio; NC, not calculated.

a "Optimal risk communication" was defined as presenting the combination of absolute risk, relative risk, and efficacy information.

b Each item in these categories is a distinct measure. The referent is the absence of the item. For example, the referent for lung cancer is stories that did not discuss lung cancer.

c A dummy variable was computed, comparing reports of new research to all other story formats.
